# Spatio-temporal analysis of malaria vectors in national malaria surveillance sites in China

**DOI:** 10.1186/s13071-015-0741-5

**Published:** 2015-03-07

**Authors:** Ji-Xia Huang, Zhi-Gui Xia, Shui-Sen Zhou, Xiao-Jun Pu, Mao-Gui Hu, Da-Cang Huang, Zhou-Peng Ren, Shao-Sen Zhang, Man-ni Yang, Duo-Quan Wang, Jin-Feng Wang

**Affiliations:** College of Forestry, Beijing Forestry University, Beijing, 100083 China; Center of 3S Technology and Mapping, College of Forestry, Beijing Forestry University, Beijing, 100083 China; LREIS, Institute of Geographic Science and Natural Resource Research, Chinese Academy of Sciences, Beijing, China; National Institute of Parasitic Diseases, Chinese Center for Disease Control and Prevention, WHO Collaborating Center for Malaria, Schistosomiasis and Filariasis, Key Laboratory of Parasite and Vector Biology, Ministry of Health, Shanghai, People’s Republic of China; Geospatial Information Sciences in the School of Economic, Political and Policy Sciences at The University of Texas in Dallas, Richardson, USA; Key Laboratory of Surveillance and Early Warning on Infectious Disease, Chinese Center for Disease Control and Prevention, Beijing, 102206 China; School of Geographical Science, Northeast Normal University, Changchun, 130024 China

**Keywords:** Malaria vectors, Spatio-temporal distribution, Cluster, Surveillance sites

## Abstract

**Background:**

To reveal the spatio-temporal distribution of malaria vectors in the national malaria surveillance sites from 2005 to 2010 and provide reference for the current National Malaria Elimination Programme (NMEP) in China.

**Methods:**

A 6-year longitudinal surveillance on density of malaria vectors was carried out in the 62 national malaria surveillance sites. The spatial and temporal analyses of the four primary vectors distribution were conducted by the methods of kernel k-means and the cluster distribution of the most widely distribution vector of *An.sinensis* was identified using the empirical mode decomposition (EMD).

**Results:**

Totally 4 species of *Anopheles* mosquitoes including *An.sinensis*, *An.lesteri, An.dirus* and *An.minimus* were captured with significant difference of distribution as well as density. *An. sinensis* was the most widely distributed, accounting for 96.25% of all collections, and its distribution was divided into three different clusters with a significant increase of density observed in the second cluster which located mostly in the central parts of China.

**Conclusion:**

This study first described the spatio-temporal distribution of malaria vectors based on the nationwide surveillance during 2005–2010, which served as a baseline for the ongoing national malaria elimination program.

## Background

Malaria, HIV/AIDS and tuberculosis (TB) are considered to be the three major public health problems in the world. In order to timely monitor the malaria epidemic and provide evidence for strategies development as well as evaluation of control activities, China government issued in 2005 the malaria surveillance program which was trialed out until 2010, and totally 62 townships in 18 provinces were selected as the sentinel sites where the malaria vectors were surveyed every 15 days from June to October [1]. However, the spatio-temporal information of continuous vectors surveillance has not been mined particularly for the NMEP from the perspective of vector control.

With continuous reduction of malaria incidence, a strong political commitment was made by China through issuing the National Action Plan for Malaria Elimination (2010–2020) in 2010. The goal is to eliminate locally acquired malaria by the end of 2015 except for the bordering areas in Yunnan Province, and eliminate the disease by 2020 nationwide. Though well-described relationships between the parasite, man and vector will provide an empirical basis for eradication of this disease, until recently there has been a paucity of systematic data on malaria vectors distribution in the malaria epidemic-prone regions of China. Undoubtedly, the efficiency of malaria elimination interventions will depend largely on information of the distribution of primary vectors. It is evident from a series of studies in China as well as in other countries [2-7] that the temporal and spatial distribution of malaria vectors is very dynamic. Thus, knowledge of the spatio-temporal malaria vector distribution will facilitate the malaria elimination efforts and overall mapping is needed to focus on priority areas for additional surveillance and response.

Therefore, the purpose of this study is to map the spatio-temporal distribution of malaria vectors to provide a dataset that would aid informed decision making on malaria elimination program, and to provide a basis for implementation of the elimination strategy in China.

## Methods

### Study areas

**Figure 1:**
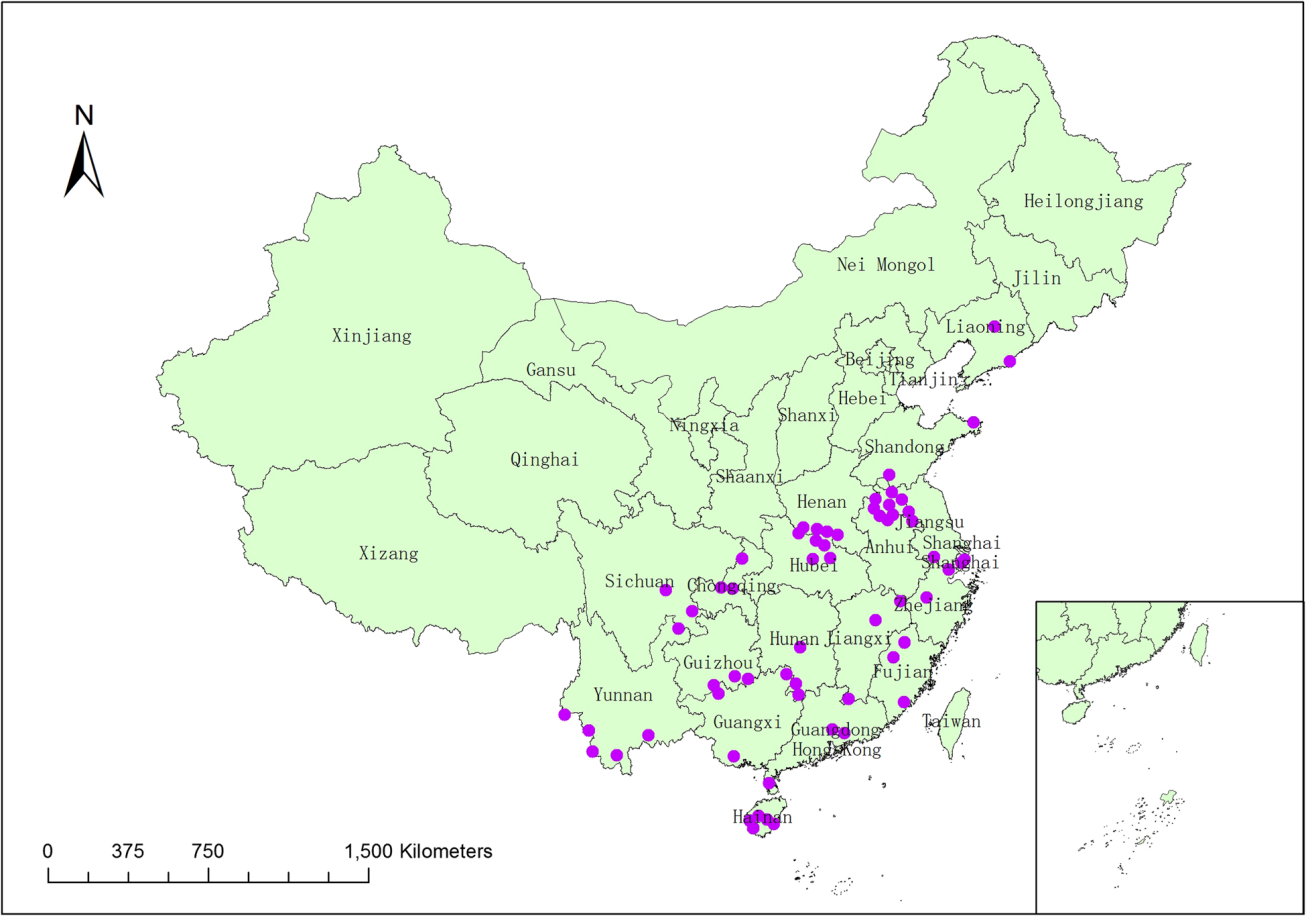
**The national malaria surveillance sites in 2005**–**2010 of China, and the purple points represent the 62 national malaria surveillance sites.**

All the 62 national malaria surveillance sites between 2005 and 2010 in China were enrolled for malaria vectors surveillance during the transmission seasons (Figure 1). These sites were divided into the following three categories based on the transmission levels of malaria [1]:

1. Unstable endemic areas: 30 townships in 6 provinces (Hainan, Yunnan, Anhui, Hubei, Henan, Jiangsu) [5 counties/ province, 1 township/county].
2. Low endemic areas: 24 townships in 8 provinces (Sichuan, Chongqing, Guizhou, Guangdong, Guangxi, Hunan, Jiangxi, Fujian) [3 counties/province, 1 township/county].
3. Pre-elimination areas: 8 townships in 4 provinces (Shanghai, Zhejiang, Shandong, Liaoning) [2 counties/province, 1 township/county].

### Malaria vector surveillance methods

According to WHO recommendations [8], human landing catches were made by two adult volunteers from the local population working beside the bed-net with one sleeping person. Mosquitoes coming to bite the collectors or sleeping person were detected using a flashlight, collected with glass tubes (CDC backpack aspirator: John W. Hock Co., Florida, USA) and placed in the screened pint-sized containers. Collections were conducted from 18:00 to 06:00 overnight during every 15 days from June to October, 2005–2010. Collectors worked in pairs for 6-h shifts. Mosquitoes were taken to the laboratory and killed by suffocation with chloroform vapor. They were counted and identified morphologically using taxonomic keys [9], and the density was calculated as the number of female adults per man per night.

### Spatial and temporal clustering

Kernel k-means method was used to identify the spatial distribution of malaria vectors in terms of density in the surveillance sites. We had surveillance on malaria vectors every 15 days during June and October from 2005 to 2010. For the 62 sentinel sites, each had 60 time series data equaling 60 dimensions. In this research, each time series data denotes the density of *An.sinensis*, *An.lesteri*, *An.dirus* or *An.minimus* per site. The time series data of these surveillance sites were interlocked, indicating the observed data in one site would not always be higher or lower than that in other sites. The classic classifier (such as k-means) would not perform well in this situation. Kernel k-means method performs well for those high dimensional and nonlinear data [10]. Comparing to the classic k-means, all of the operations will be done in feature space for kernel k-means [11]. With kernel k-means method, all data are projected in high dimension feature space that the data will be easily linearly classified in feature space. In feature space, the original data (*x*_1_,…,*x*_N_) which represent the density of the malaria vector, will be projected to be (*φ*(*x*_1_), …, *φ*(*x*_*N*_)). The processes of kernel k-means are as follows [10]:

- Initializing the centers *m*_*k*_(*k* =1,…,*K*) for those clusters in feature space.
- Assigning each observed value *φ*(*x*_*i*_)(*i* = 1, …, *N*) to the nearest center, and the distance between observed value and center is computed through Euclidean distance:
  $$ d= \arg \min \left\Vert \varphi \left({x}_i\right)\right.-{\left.{m}_k\right\Vert}^2 $$
- Update the centers and recalculate the sum of the within-cluster variation E;
  $$ {m}_k=\frac{1}{N_k}{\displaystyle \sum_{x_j\in classk}\varphi \left({x}_j\right)},E={{\displaystyle \sum_{k=1}^K{\displaystyle \sum_{j=1}^{N_k}\left\Vert \varphi \left({x}_j\right)-{m}_k\right\Vert}}}^2 $$
- Repeat step (b) and (c), until the E is stable or less than a threshold value.

Caliński [12] proposed the *CH* index to indicate the number of clusters. The *CH* index simultaneously includes the variances of within-cluster and between-clusters. The formula of *CH* index is as follows:

$$ CH(k)=\left[B/\left(k-1\right)\right]/\left[W/\left(n-k\right)\right] $$

*B* denotes variances of between-clusters, *W* are the variances of within-cluster, k is the clusters’ count, *n* indicates the count of monitoring station. The high *CH* value indicates the well clustering results. In this research, we chose the count of clusters when the *CH* index is highest. The kernel k-means function was used the “kern lab” package [13] in R [14] in our study.

### Temporal period and trend decomposition

Empirical mode decomposition (EMD), an adaptive time series data analysis model [15,16], was used to identify the temporal trend of malaria vectors in terms of density in the clustered surveillance sites. In EMD analysis, the original data is decomposed into a series of modes without requiring prior knowledge. Comparing with Fourier and wavelet decomposition, EMD has its own advantage. The Fourier analyses could transform data into the combination of sine and cosine functions with different frequencies, while wavelet analysis needs wavelet splines. These types of decomposition involve many spurious components due to serious restriction of the harmonic nature of the basis function [10,17]. The EMD method decomposes the data into several oscillatory components, corresponding to some physical phenomenon underlying the data, and the residual of the decomposition is the trend of the data [10].

The model decomposed from EMD methods is called the intrinsic mode function (IMF) [15,16]. An IMF should satisfy two conditions [10,15]: First, the difference between the number of extrema and the number of zero-crossings in the whole data span should be less than or equal to one. Second, the mean value of the envelope defined by the local maxima and the envelope defined by the local minima is zero at any point. We calculated the IMFs and the residuals of the density of the malaria vectors using the sifting method by taking the following steps:

Initialize *r* (*t*) = *x*(*t*), *i* = 0, *k* = 1, where *x*(*t*) is the time series of density of the malaria vectors. We define a threshold *δ* , the count of extreme points *N*.

Step1: Calculate the local maximum value and the local minimum value of the time-series *r*(*t*);

Step2: Calculate the upper envelope *e*_max_(*t*) through interpolating the local maximum value, and the lower envelope *e*_min_(*t*) through interpolating the local minimum value;

Step3: Calculate the local average value using the formula: *m*(*t*) = (*e*_max_(*t*) + *e*_min_(*t*))/2;

Step4: Appoint *i* = *i* + 1, then the proto-mode function (PMF) is calculated through the following formula: *p*_*i*_(*t*) = *r*(*t*) − *m*(*t*), *r*(*t*) = *p*_*i*_(*t*);

Step 5: The termination condition is:

$$ SD={\displaystyle \sum_t\left[\frac{\left|{p}_i(t)-{p}_{i-1}(t)\right|{}^2}{{p^2}_{i-1}(t)}\right]} $$

If SD < *δ*, then *imf*_*k*_(*t*) = *p*_*i*_(*t*), go to the following step; otherwise, go back to step 1–4;

Step 6: Appoint *r*(*t*) = *r*(*t*) − *imf*_*k*_(*t*). If the count of extreme points of *r*(*t*) is greater than *N*, then *k* = *k* + 1, *i* = 0, and go back to step 1; otherwise, stop the sifting process.

The density series of malaria vectors *x*(*t*) can then be expressed as:

$$ x(t)={\displaystyle \sum_{i=1}^Kim{f}_i+r} $$

Where *K* is the number of IMFs and *r* is the trend of the original data. The first IMF (IMF1) is the component with the highest frequency. All of the IMFs were statistically tested against the null hypothesis [18]. In this research, the EMD method was processed with the code written by Rilling et al. [19]

## Results

### Overview of the dataset

**Figure 2:**
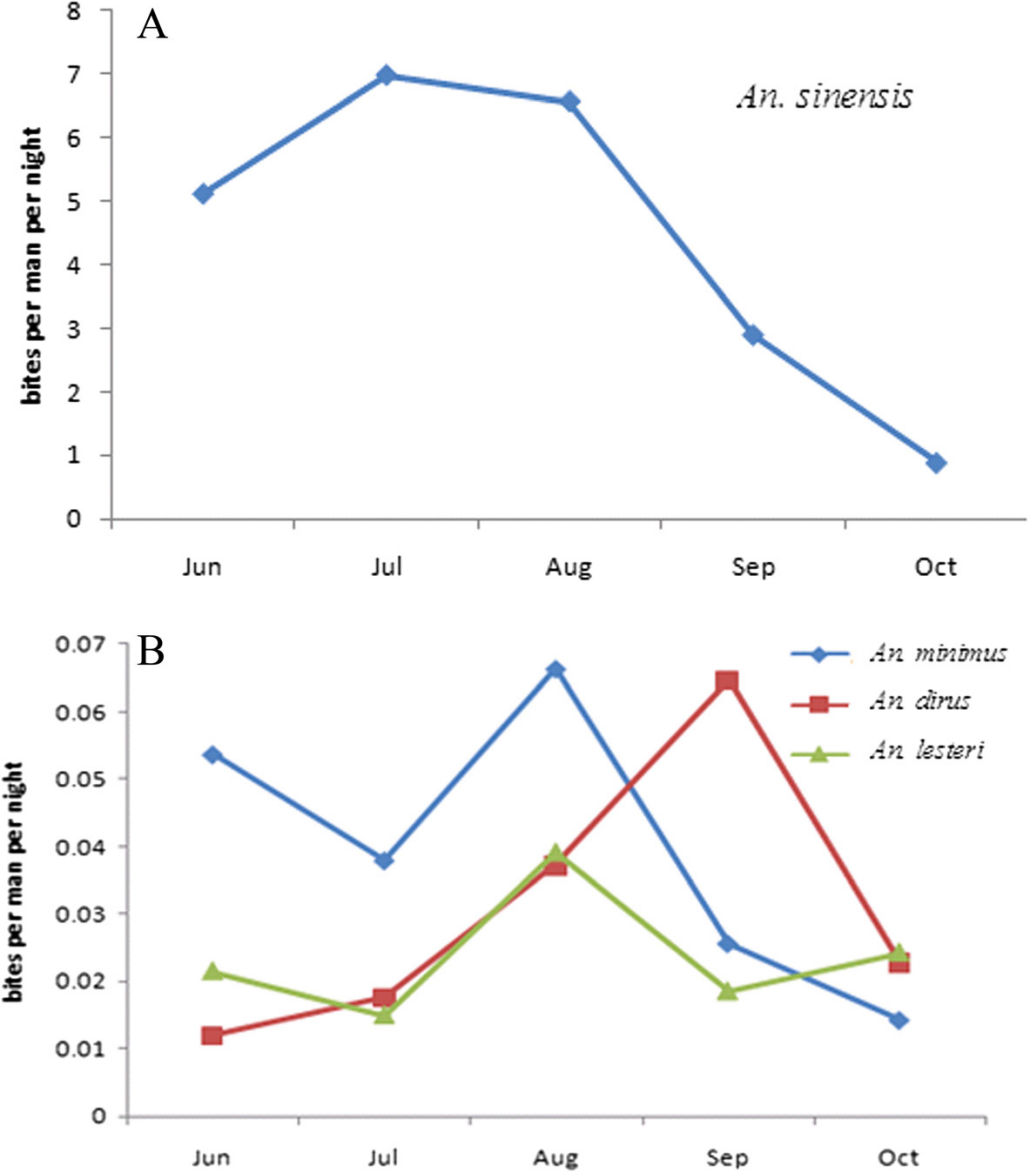
**Seasonal distributions of An.sinensis (A) and An.lesteri, An. dirus and An. minimus (B) in the surveillance sites during 2005–2010.**

A total of 37,257 female anopheles were captured in the 62 surveillance sites during 3,720 nights of collecting from 2005 to 2010, with a significant difference in the distribution as well as density of different species. The collections contained 4 species namely *An.sinensis* representing 96.25% (35,861/37,257), *An. lesteri* 0.75% (278/37,257), *An.dirus* 1.49% (555/37,257) and *An.minimus* 1.51% (563/37,257). The above observations indicated the most widely distribution of *An. sinensis* in the all surveillance sites (62 townships) of 18 provinces, but showed great variation. It had a classic peak of abundance each year in the rainy season (July-August), while the other malaria vectors had the peak of abundance during August-September in the surveillance sites during 2005–2010 (Figure 2).

**Figure 3:**
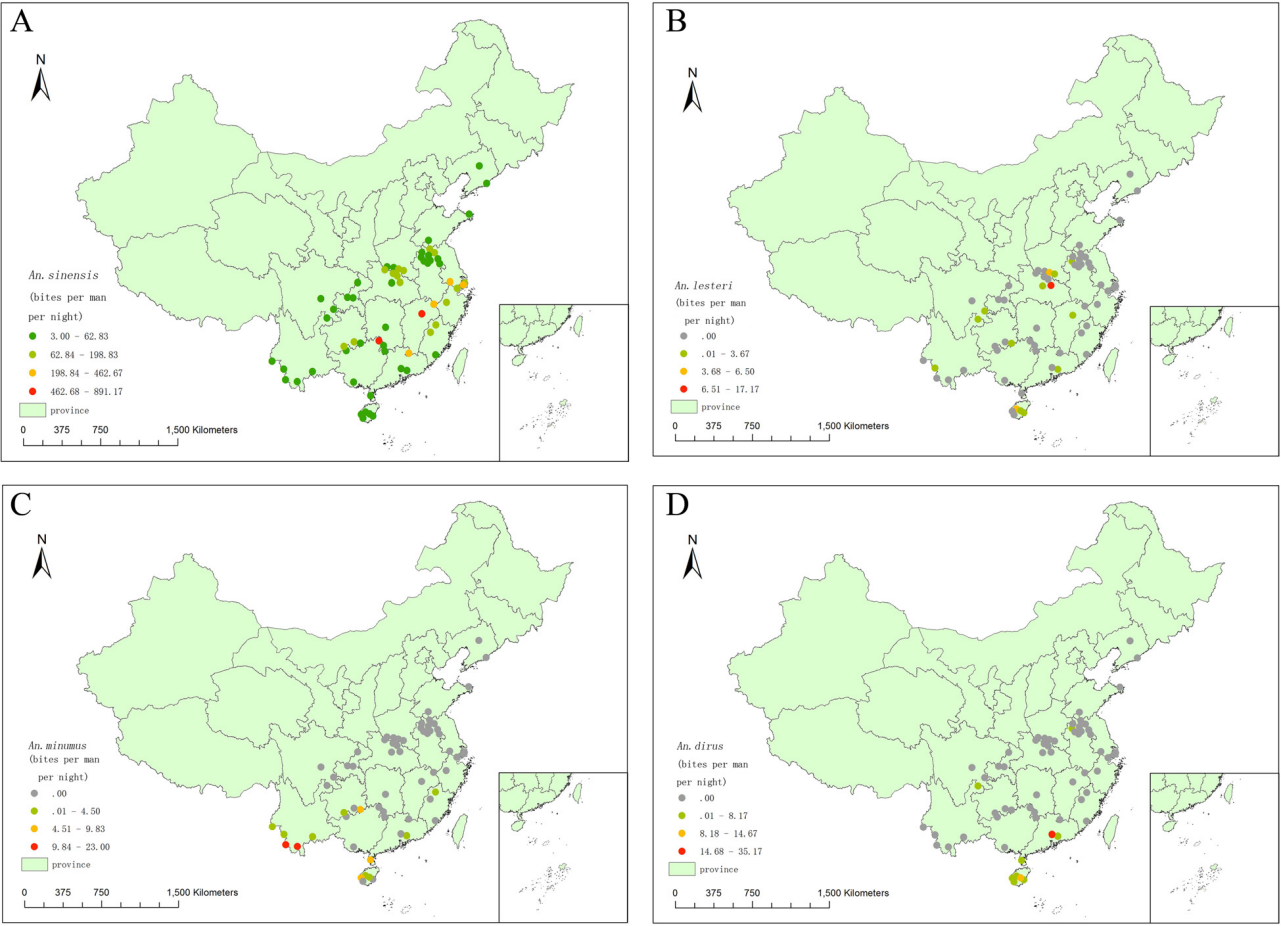
**The spatial distribution of malaria vectors in the surveillance sites of China during 2005–2010.** (**A**. *An. sinensis*; **B**. *An. lesteri*; **C**. *An. minimus*; **D**. *An. dirus*).

The density of *An. sinensis* per site averaged 6.21 and ranged between 0.40 and 107, the three highest density of *An. sinensis* was observed at Quanzhou site (106.94 bites per man per night) in Guangxi Province, followed by Jinxian (71.57 bites per man per night) and Wuyuan (54.43 bites per man per night) in Jiangxi Province, while the three lowest density of *An. sinensis* was found at Jiangyang (1.06 bites per man per night), Qingshen (0.49 bites per man per night) and Junlian (0.40 bites per man per night) in Sichuan Province (Figure 3A).

*An. lesteri* was found at 13 surveillance sites in 7 provinces (Henan, Hubei, Guangdong, Hainan, Sichuan, Guizhou and Yunnan), with a peak of abundance each year from June to July. The density per site averaged 0.45 and ranged from 0.03 to 2.29 bites per man per night, the three highest density was observed at Anlu (2.29 bites per man per night) in Hubei Province, followed by Tongbai (0.80 bites per man per night) in Henan Province and Baisha (0.78 bites per man per night) in Hainan Province (Figure 3B).

*An. minimus* was captured at 13 national malaria surveillance sites in 5 provinces (Fujian, Guangdong, Hainan, Guizhou and Yunnan),with a peak of abundance each year from July to September. The density of *An. minimus* per site averaged 1.26 and ranged from 0.20 to 3.57 bites per man per night, the two highest density was observed at Menglian (3.57 bites per man per night) and Jinghong (3.50 bites per man per night) from Yunnan Province (Figure 3C).

*An. dirus* was captured at 8 national malaria surveillance sites in 3 provinces (Guangdong, Guizhou and Hainan). The density of *An. dirus* per site averaged 1.21 and ranged between 0.11 and 4.22 bites per man per night, the highest density was observed at Zengcheng (4.22 bites per man per night) in Guangdong Province, followed by Congjiang (1.76 bites per man per night) in Guizhou Province (Figure 3D).

### Clustering distribution

**Figure 4:**
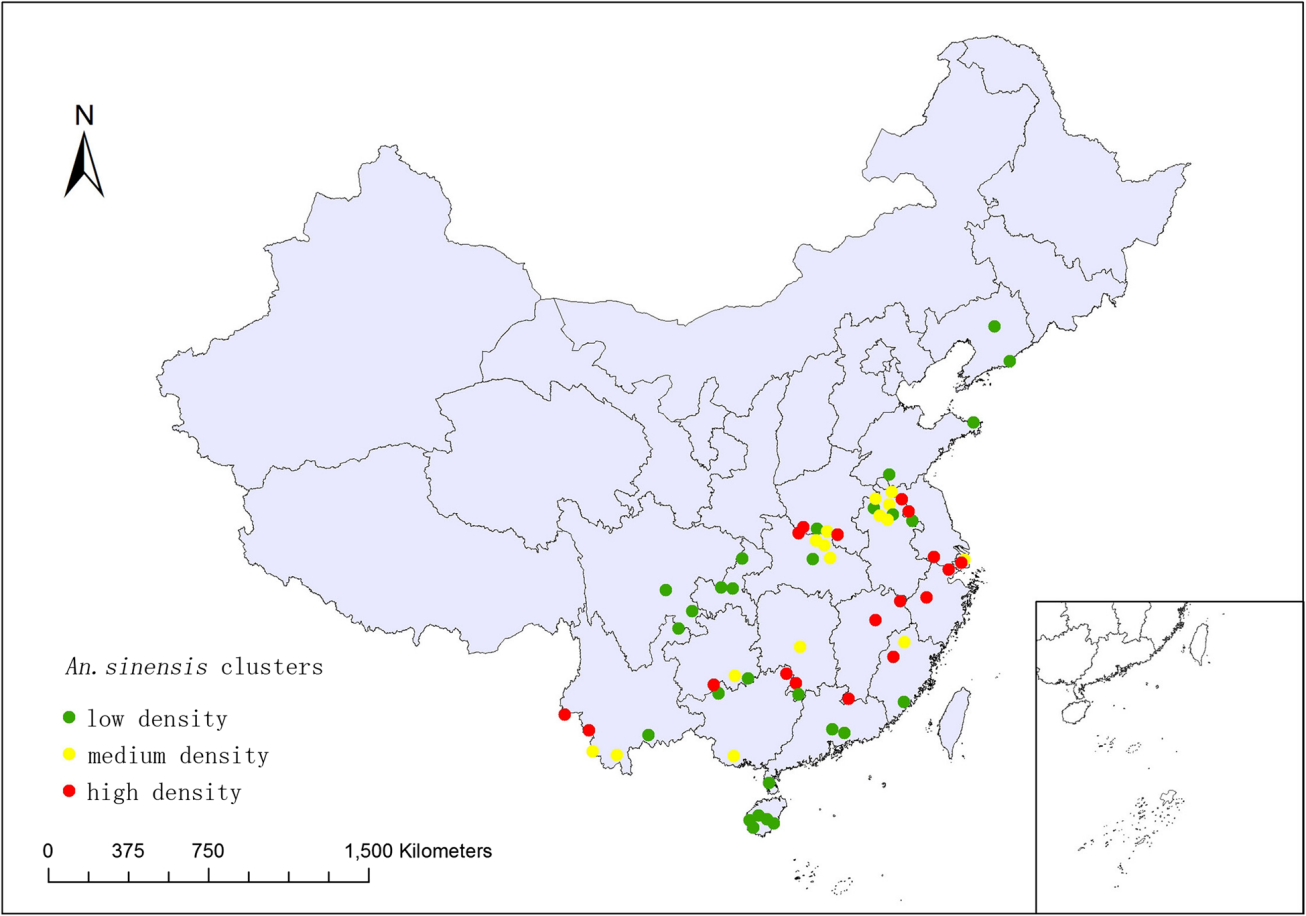
**The cluster distribution of** ***An. sinensis*** **in the surveillance sites during 2005–2010.**

Since the density distribution of *An. sinensis* in some sites was similar in the surveillance regions, kernel k-means was used to identify these similarities, and three distinct clusters were determined among the surveillance regions based on CH value [10] (Figure 4).

The first cluster (low *An. sinensis* density) including 28 sentinel sites in 18 provinces was the most widely distributed with the lowest median density of 0.48 bites per man per night comparing to the other two clusters, the second cluster (medium *An. sinensis* density) was mainly concentrated mostly in the central parts of China with the median density of 1.67 bites per man per night, especially in Hubei and Anhui Provinces, the third cluster (high *An. sinensis* density) was centralized in the southeastern part of China such as Fujian, Zhejiang and Jiangxi Provinces with highest median density of 5.03 bites per man per night (Table 1).

**Table 1:** **The density distribution in three clusters of** ***An. sinensis*** **during 2005-2010**

**Clusters**	**Mean(SD)**	**Minimum**	**Percentile**			**Maximum**
			**25%**	**50%**	**75%**	
Low density	0.21	0.05	0.29	0.48	0.58	0.87
Medium density	0.95	0.16	0.83	1.67	2.26	4.24
High density	4.00	0.29	2.38	5.03	7.56	17.20

### The temporal trend of identified clusters

**Figure 5:**
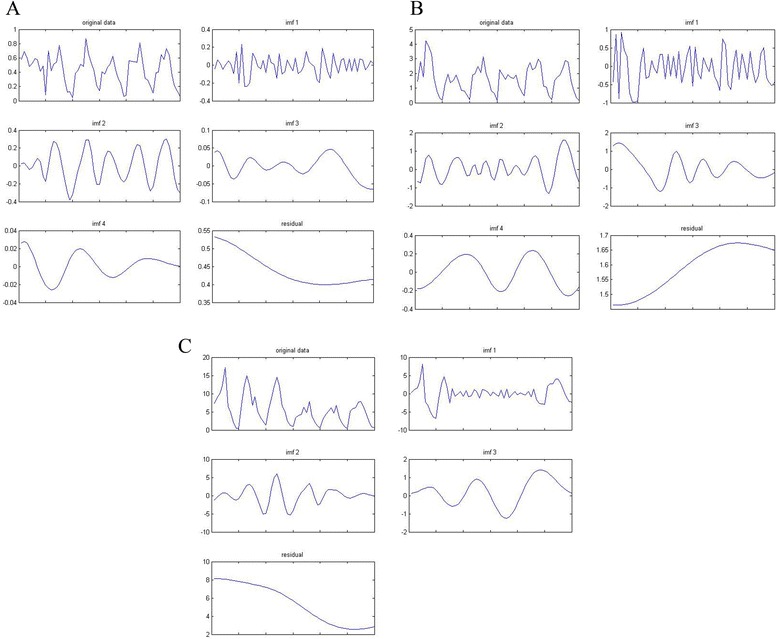
**EMD decomposition of the three clusters of** ***An. sinensis*** **density: (A) first cluster (low density) (B) second cluster (medium density) (C) third cluster (high density).**

During the surveillance period, a significant decrease in *An. sinensis* density was found in the first as well as the third cluster. In the first cluster, most decrease in the density (from 0.53down to 0.39 bites per man per night) occurred during 2005–2008 with little change between 2009 and 2010, while the decrease was significant (from 8.1 to 2.5 bites per man per night) in the third cluster during the total surveillance period. However, an increase of *An. sinensis* density was observed in the second cluster during whole surveillance period with a significant increase (from 1.4 to 1.7 bites per man per night) during 2006–2009, while a little decrease in 2010, which was a big difference from the other two clusters (Figure 5).

## Discussion

Like many former studies [20-25] indicating four primary malaria vector species including *An.sinensis, An.lesteri, An.dirus* and *An.minimus* existed in China, this study also showed the same malaria vectors existing with significant discrepancy of spatial distribution in the surveillance regions.

Although *An.sinensis* is an inefficient vector mainly because of its zoophilic habit, it is still considered an important vector of *P. vivax* malaria due to its wide distribution as well as high density. Malaria transmission had often taken place due to its high density under suitable conditions such as the open plains in the greater part of China between 25 ~ 33 north latitude [25-27].

*An. lesteri* was mainly distributed in low hill, hillock and shallow hilly plain between 22 ~ 33 north latitude which had still been found in 11 provinces with 205 counties through the survey conducted from 1998 to 2001 [3]. However, *An. lesteri* had been captured only in 13 counties from 7 provinces (Henan, Hubei, Guangdong, Hainan, Sichuan, Guizhou and Yunnan) in the surveillance sites during 2005–2010, indicating that the spatial distribution of this vector became smaller than before. The extensive agricultural use of pesticides and indoor insecticide spraying may have substantially reduced its distribution [23,28,29].

*An.minimus* had long been regarded as a principal malaria vector in China. In the past, mosquitoes of the *An. minimus* group were recorded from 16 provinces in southern China including Sichuan, Chongqing, Hubei, Henan, Anhui, Zhejiang, Yunnan, Guizhou, Guangxi, Hainan, Hunan, Jiangxi, Guangdong, Fujian, Hong Kong and Taiwan throughout southern China from Yunnan Province eastward and from Hainan Island northward to approximately 32.5 north latitude [30-33]. However, *An. minimus* was captured only in 5 provinces (Fujian, Guangdong, Hainan, Guizhou and Yunnan) from the surveillance sites during 2005–2010. It is still a major malaria vector in the hilly bordering regions of Yunnan Province near Myanmar [33].

In China, *An.leucosphyrus* was found for the first time by the writer in 1941 in Yunnan [34]. It was then rectified to *An.dirus*, a new species described by Peyton and Harrison [35], based on the detailed morphological comparison of specimens from Thailand. However, different from previous research that *An.dirus* had been captured from four provinces of Hainan, Yunnan, Guangxi and Tibet [36], it was only captured at 8 national malaria surveillance sites during 2005–2010 from 3 provinces (Guangdong, Guizhou and Hainan). *An.dirus* has so far been known as a malaria vector only on Hainan Island.

Further, this is the first study to systematically explore the temporal-spatial dynamics of *An.sinensis* in China using kernel k-means based on CH value [10]. The significant difference of population density may have resulted from available resources that more rice fields in the southern part of China than the others contributing to the suitable breeding sites of *An.sinensis* [37]. Different from the first and third cluster that a significant decrease of *An. sinensis* density was observed, the second cluster displayed a significant increase during 2005–2010 in the central part of China, which the increasing vectorial capacity leading to malaria re-emergence in Huang-Huai River of central China may be related to this change [38,39].

## Conclusion

As malaria transmission relies mainly on *Anopheles* mosquitoes, the present results will serve as a baseline for the ongoing NMEP in China, which will help to address the knowledge gaps with respect to the malaria profile under different settings.
